# Preparation of hexagonal nanoporous Al_2_O_3_/TiO_2_/TiN as a novel photodetector with high efficiency

**DOI:** 10.1038/s41598-021-96200-2

**Published:** 2021-09-02

**Authors:** Asmaa M. Elsayed, Mohamed Rabia, Mohamed Shaban, Arafa H. Aly, Ashour M. Ahmed

**Affiliations:** 1 Nanophotonics and Applications (NPA) Lab, Physics Department, Faculty of Science, Beni-Suef University, Beni Suef, 62514 Egypt; 2 TH-PPM Group, Physics Department, Faculty of Science, Beni-Suef University, Beni Suef, 62514 Egypt; 3 Polymer Research Laboratory, Chemistry Department, Faculty of Science, Beni-Suef University, Beni Suef, 62514 Egypt; 4 Department of Physics, Faculty of Science, Islamic University of Madinah, P. O. Box: 170, Al Madinah Almonawara, 42351 Saudi Arabia

**Keywords:** Energy science and technology, Nanoscience and technology

## Abstract

The unique optical properties of metal nitrides enhance many photoelectrical applications. In this work, a novel photodetector based on TiO_2_/TiN nanotubes was deposited on a porous aluminum oxide template (PAOT) for light power intensity and wavelength detection. The PAOT was fabricated by the Ni-imprinting technique through a two-step anodization method. The TiO_2_/TiN layers were deposited by using atomic layer deposition and magnetron sputtering, respectively. The PAOT and PAOT/TiO_2_/TiN were characterized by several techniques such as X-ray diffraction (XRD), scanning electron microscope (SEM), and energy dispersive X-ray (EDX). The PAOT has high-ordered hexagonal nanopores with dimensions ~ 320 nm pore diameter and ~ 61 nm interpore distance. The bandgap of PAOT/TiO_2_ decreased from 3.1 to 2.2 eV with enhancing absorption of visible light after deposition of TiN on the PAOT/TiO_2_. The PAOT/TiO_2_/TiN as photodetector has a responsivity (R) and detectivity (D) of 450 mAW^-1^ and 8.0 × 10^12^ Jones, respectively. Moreover, the external quantum efficiency (EQE) was 9.64% at 62.5 mW.cm^−2^ and 400 nm. Hence, the fabricated photodetector (PD) has a very high photoelectrical response due to hot electrons from the TiN layer, which makes it very hopeful as a broadband photodetector.

## Introduction

Recently, optical detections have highly research interest in many fields such as biological detection, industrial automation, environmental monitoring, and space exploration^1–3^. Moreover, these semiconductor metal oxides or polymer have other applications in photocatalytic water splitting and solar cell studies^4–6^. The desirable photodetector devices in industrial applications must have high performance include the fast response, high sensitivity, easy operation, and low^4–6^ power consumption^7^. Many previous studies focused on the preparation of PD for light in the UV region depended on using photoactive materials that can work in these regions like GaN, ZnO, and SiC^8,9^. These materials can accept new properties for light detection through increasing the active sites in nanowires or nanotubes structures^10,11^.

The plasmonic materials increase the efficiency of the photodetectors due to the unique properties of the induction surface plasmonic resonance results from the oscillation of the higher electrons in the conduction band^12^. Plasmonic resonance improve the electrical and optical material properties by enhancement the electric field of the incident light by several magnitude^13,14^. The most used plasmonic materials are noble metals such as Au, Ru, Rh, and Pt. Unfortunately, these metals are very expensive^15^. At the same time, many researchers used active metals such as Co, Fe, Ni, and Cu as plasmonic materials. Li et al. used Cu nanomaterials to enhance the light capture and efficiency for the light detection in the Cu/ZnO photodetector^16^. Although these elements are cheap, they are active and they easy to form oxides.

On the other side, metal nitrides such as TiN and ZrN acting as photo plasmonic materials for light capture. Moreover, these metal nitrides used in designing devices with spectral windows and operating conditions more efficient in hazardous environmental conditions than the noble metals^17^. Surre et al. compared the effect of TiN and ZrN plasmonic nanomaterials with noble metals on sensor plasmon refractometer sensitivity properties^18^. The results show that the nitride metals have a high efficiency compared with the noble metals. Mohamed et al. used the TiN layer for enhancing the properties of TiON photoactive material for solar cell using the good TiN properties; hardness, nontoxicity, high thermal conductivity, high melting point, good photochemical stability, and high UV–Vis light absorbance^19^.

Besides, TiN is characterized by a catalytic effect that enables it to be used in self-cleaning. Awad et al. used triple layers from TiO_2_/TiN/TiO_2_ for self-cleaning^20^. The optical properties of the prepared layers are enhanced with increasing TiN thickness which facilitates the degradation of organic dye. Moreover, the TiN was used in devices such as fuel cells, supercapacitors, and solar cells^21–23^.

On the other side, TiO_2_ nanomaterial with a high surface area has a special interest in photocatalytic application^24^. Nanotubes accept a more active site from the internal or external tube with a large surface area per volume. Also, TiO_2_ has additional good properties such as biocomPAOTibility, low-cost, easy preparation, and high stability^23^. These properties qualify TiO_2_ materials for applying in sensors, supercapacitor, photodegradation, solar cell, and light absorbance^25^. Kunwar et al. studied the GaN/TiO_2_/Au layers to increase the light detection region to reach the visible region^1^. Another study based on TiO_2_-graphene for enhancement of the UV photodetection was carried out, the incorporation of graphene to increase the light absorbance region^24^.

On the other hand, PAOT has a highly ordered two-dimensional hexagonal porous structure with high surface area which improves the interaction with light. Also, PAOT has high stability (chemically, thermally, and mechanically), good optical properties, biocomPAOTible, abundant, and inexpensive. PAOT is an attractive material as template for fabricate of nanotubes, nanowires, and nonodotes arrays^26^. This because its unique nanometric properties which are highly required in potential broad applications including biosensors, catalysts, magnetic storages, solar cells, optoelectronics, photonic crystals, and drug delivery systems^27^. PAOT structure is a typical self-ordered nanoporous material composed of hexagonally arranged cells with cylindrical pores in the centers that are aligned perpendicular to Al surface^28,29^. These hexagonal PAOT shapes can be easily transferred to other materials by depositing the material into the pores of the PAOT using some techniques such as chemical vapor deposition, atomic layer deposition, or magnetron sputtering deposition. The used technique can fabricate arrays of nanophotonic structures. The dimensions of the nanophotonic structures can be controlled by changing the geometrical structure of the PAOT.

In this study, we have prepared a novel PAOT/TiO_2_/TiN PD with high stability and efficiency. All the materials of the photodetector were prepared with high controlled technique. PAOT was prepped by the Ni-imprinting method, then TiO_2_/TiN deposition occurred using atomic layer deposition and direct current sputtering techniques, respectively. The application of the PAOT/TiO_2_/TiN as a photodetector occurred by study the effect of light power intensity and wavelengths. The photodetector parameters R, D, and EQE were determined. The prepared photodetector can be applicable in the industrial field with high stability and low cost.

## Experimental part

### Aluminum oxide template synthesis

The pores aluminum oxide template (PAOT) was prepared using the imprinting technique using Ni-mold through two step anodization method^23^. Ni mold has a hexagonal nanopillar order with 400 nm space shallow array. An electropolishing process for the Al foil was completed using solution C_2_H_5_OH and HClO_4_ (1:1). The imprinted Al foil (99.99%) was obtained by applying 10 kN/cm^2^ using an oil pressing system for 3 min. After that, the two-steps anodization process occurred at 160 V in ethylene glycol:H_3_PO_4_:H_2_O (100:200:1) electrolyte at 2 °C for 15 and 120 min, respectively. After the first anodization, the chemical etching process was carried out using H_3_PO_4_ (6 wt%) and H_2_CrO_4_ (1.5 wt%) mixture at 60 °C for 12 h. Finally, the pores widening process occurred through immersing the PAOT into H_3_PO_4_ (6 wt%) solutions for complete synthesis of the Al_2_O_3_ template with hexagonal pores.

### Synthesis of TiO_2_/TiN

TiO_2_/TiN nanotube composite was prepared inside the PAOT by using atomic layer deposition and magnetron sputtering physical devices, respectively. TiO_2_ nanotube composite was prepared inside the PAOT by using atomic layer deposition (ALD, Picosun SUNALE R150) at 300 °C. TiCl_4_ and H_2_O were used as sources for Ti and O, respectively.

TiN nanotube was fabricated inside the PAOT/TiO_2_ by using magnetron sputtering (DC sputtering, LA440S Ardenne). The sputtering was carried out in a mixture of N_2_ and Ar gases with a rate of 75 and 25 sccm, respectively. High purity Ti metallic (99.9%) was used as a target at working pressure was 13 × 10^–3^ mbar. The PAOT/TiO_2_ substrate was about 6 cm from the Ti-target at 250 °C.

### Characterization

The characterization of prepared ample was carried out by different analytical tools, in which the material morphology was proved using SEM (SEMAuriga Zeiss FIB). Moreover, the SEM device has another unit tool for energy dispersive X-ray (EDX) analysis. The chemical structure was confirmed using an X-ray diffractometer (Bruker/Siemens D5000, XRD). In addition to that, a double beam spectrophotometer device (Perkin Elmer, Lamba 950) used for the optical analyses.

### Photodetector fabrication process

**Figure 1:**
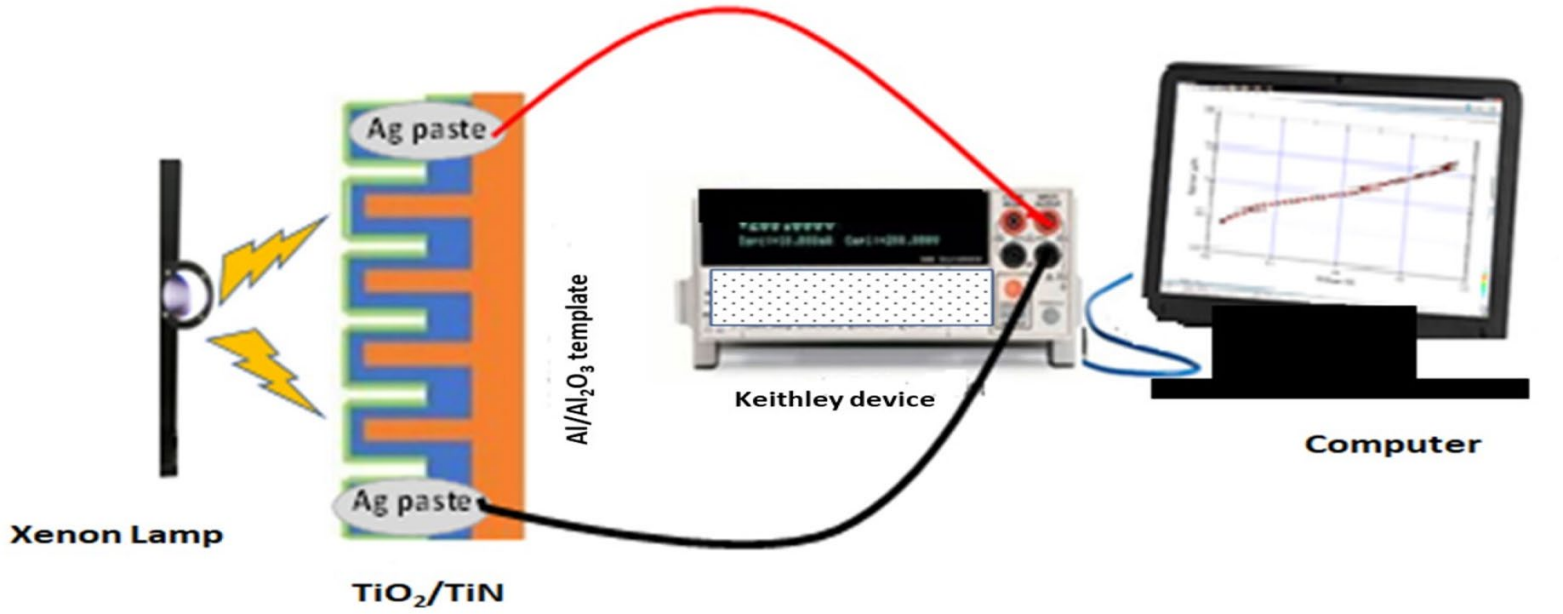
Scheme for testing the prepared materials as a photodetector.

The photodetector measurements of the prepared PAOT/TiO_2_/TiN sample were carried out using the Keithley device (model 2500, Tektronix Company) as seen in schematic Fig. 1. The measurements investigated though − 1 to + 1 V under Xe lamp (Newport) as a light source. Two electrodes were connected over the sample using a silver paste, the final size was 1 cm^2^. The effects of light power intensity and light wavelengths on the photodetector were studied. Also, the effect of sample stability under light was investigated. All measurements for the fabricated device carried out at room temperature and normal atmosphere.

## Results and discussion

### SEM and XRD analyses

**Figure 2:**
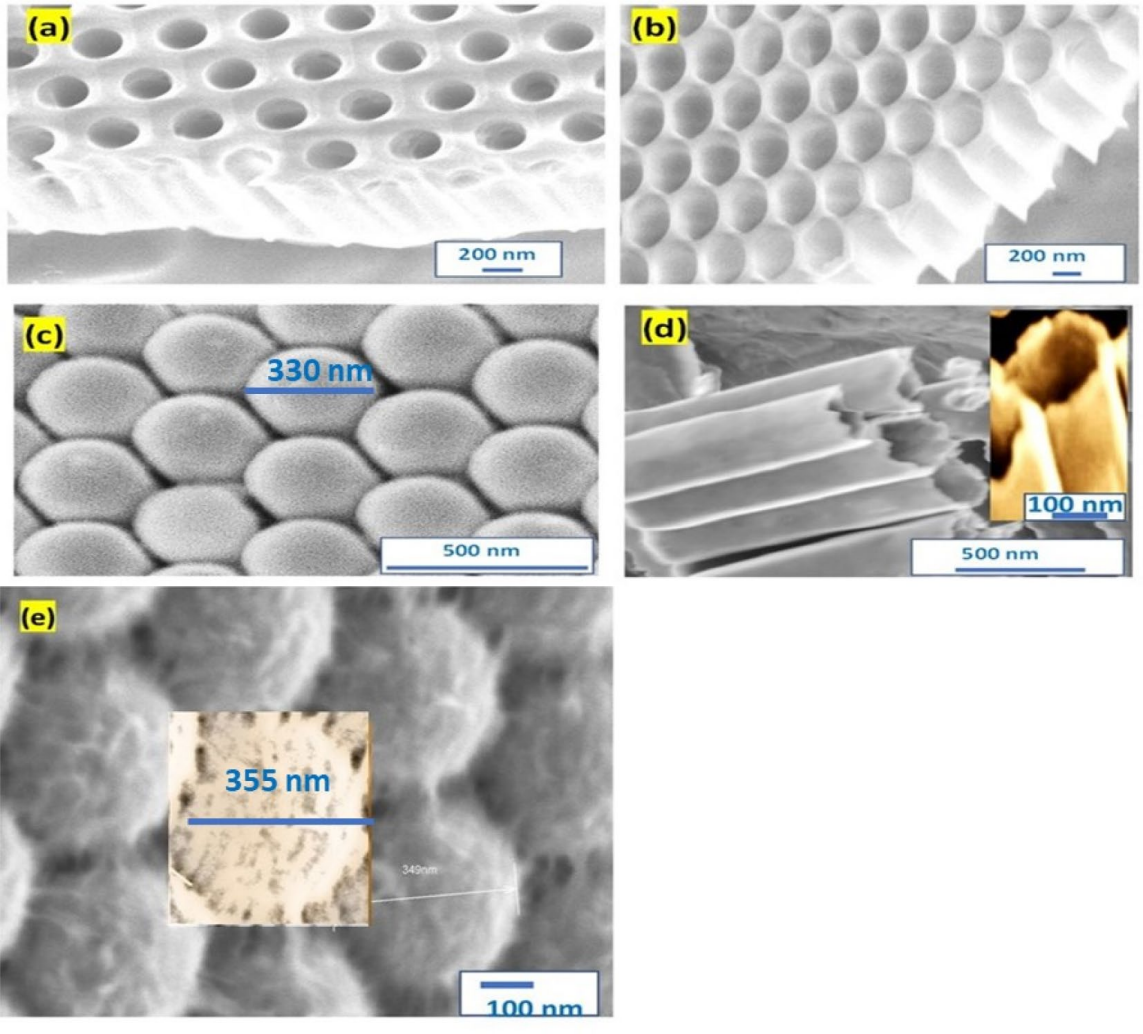
The SEM of Al_2_O_3_ template after pore widening for (**a**) 10 and (**b**) 30 min at 60 °C. (**c**) Bottom image (**d**) cross-section image of the TiO_2_/TiN hollow tubes after removing PAOT. (**e**) TiO_2_/TiN (bottom image).

The SEM of PAOT after pore widening using H_3_PO_4_ solution is shown in Fig. 2a,b for 10 and 30 min, respectively. The figure shows a highly ordered hexagonal nanoporous Al_2_O_3_ array. For pore-widening 10 min, the PAOT pores are about 245 nm with an interpore distance of about 187 nm. While after pore widening for 30 min, the PAOT pores increase to 305 nm with an interpore distance of about 61 nm as seen in Fig. 2b.

Moreover, Fig. 2c,d appears the bottom and cross-sectional views of TiO_2_/TiN nanotubes after the PAOT removal using NaOH solution. The TiO_2_ nanotubes are appearing hollow nanotubes closed from one side and arrayed on a hexagonal arrangement as same for PAOT. The TiO_2_ nanotube has wall thickness of 20 nm and length of 2.3 µm as shown in Fig. 2d. The TiN over the PAOT increases the active sites for light detections though large surface area. Moreover, the diameter of the TiO_2_ increase from 330 to 355 nm after deposition of TiN as shown in Fig. 2e.

**Figure 3:**
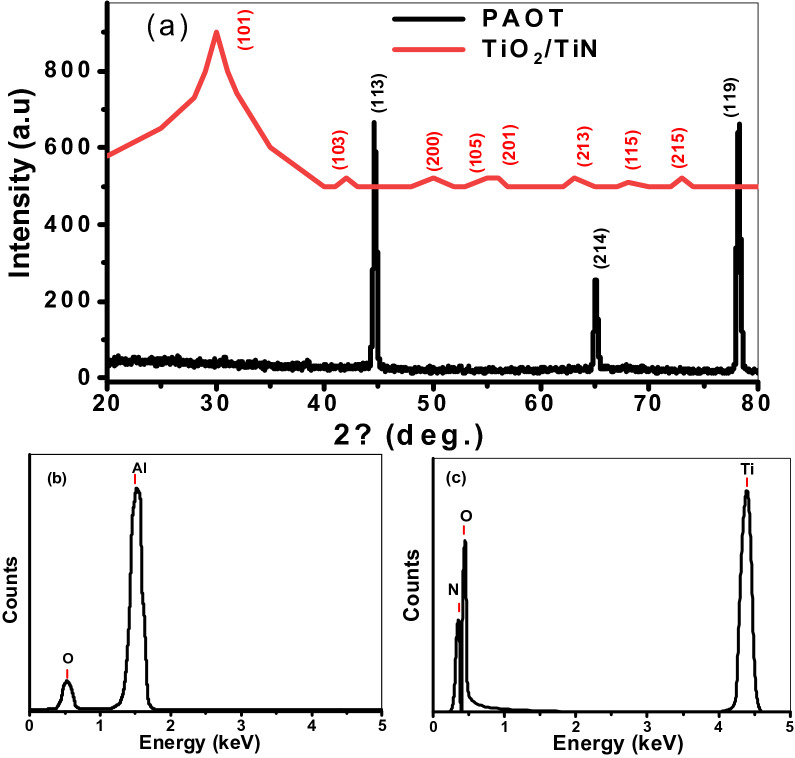
(**a**) The XRD pattern for PAOT and TiO_2_/TiN, (**b**) EDX chart of PAOT, and (**c**) EDX spectra of TiO_2_/TiN.

The XRD of PAOT and TiO_2_/TiN is mentioned in Fig. 3a. From this figure, there are three sharp peaks for the Al_2_O_3_ template are located at 44.5, 65, and 78.1° for the growth directions (113), (214), and (119), respectively (JCPDS card # 01-089-4921). The sharp-peaks indicate the Al_2_O_3_ template is crystalline and easy to fabricated using the Ni-imprinting technique^30^.

The XRD of TiO_2_/TiN nanotubes has eight peaks for the growth of TiO_2_ (anatase form), These peaks are located at angles 25.8, 38.1, 48.3, 54.3, 55.1, 62.8, 69.8, and 75.3^O^ for the growth directions (101), (103), (200), (105), (211), (213), (118), and (215), respectively.

The peaks observed for PAOT in Fig. 3a agree with previous works^31,32^. These peaks indicate that the PAOT is polycrystalline structure. The high intensity of (119) is indicating good crystallization for growth crystal in this orientation. Also, the porous alumina was well aligned perpendicular relatively to the surface of Al layer. Therefore, there was porous Al_2_O_3_ layer formed on the Al substrate surface during anodizing process.

After deposition of thin TiN over TiO_2_, no phase is created. The thin thickness of the TiN layer (8 nm) can be produced out of phase diffraction for X-ray interface between TiO2/TiN layers. This due to the N_2_ atoms may occupy the locations of O_2_ atoms in anatase TiO_2_ crystal or they are located at the grain boundaries and form amorphous portions. Also, the scattering effect of X-ray radiations can be produced out of phase diffraction at the interface between TiO_2_/TiN layers^33,34^, hence don't verified the Bragg condition. Moreover, the higher reactivity of oxygen towards titanium lead to prefer the formation of Ti–O bonds compared than Ti-N bonds and hence helps the formation of TiO_2_ phase which is thermodynamically stable phase^35^.

For confirming the elements of the prepared materials, the EDX analyses are mentioned in Fig. 3. From this figure, the elements Al and O are confirmed for the Al_2_O_3_ template (Fig. 3b). Moreover, the elements Ti, O, and N are confirmed for the nanotube TiO_2_/TiN (Fig. 3c). The quantitative results of the PAOT were 62.3% Al and 37.7% O which shows the high purity Al_2_O_3_. For the TiO2/TiN bilayer, the atomic ratio of Ti:O:N is 44.63:35.11:20.26.

### Optical analyses of the prepared materials

**Figure 4:**
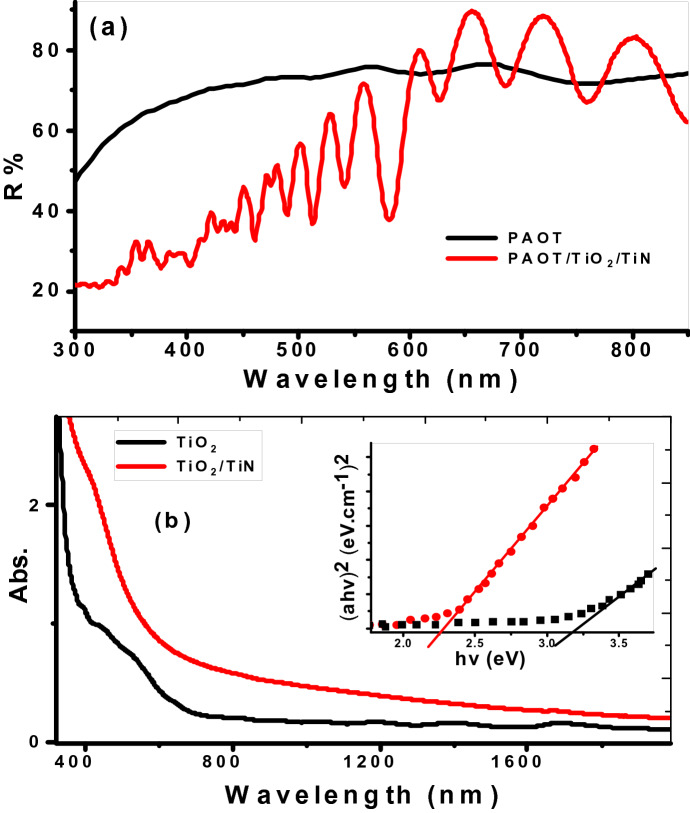
Optical properties of TiO_2_ and TiO_2_/TiN; (**a**) reflection and (**b**) absorption spectra. The inset of (**b**) shows $(\upalpha \mathrm{h}\upnu )$ ^2^ versus $\mathrm{h }\upnu$ for energy gap calculation.

The UV–Vis optical characteristic of PAOT/TiO_2_/TiN are very important for the photodetector application. The optical reflectance spectra of the PAOT and PAOT/TiO_2_/TiN are mentioned in Fig. 4a. In UV and visible regions, the average PAOT/TiO2/TiN reflectivity compared to PAOT is relatively low. This means that absorbance increases in the visible region after the deposition of TiO_2_/TiN on the template. All the spectra exhibit PAOTterns oscillations of interference fringes^30^. There are very small interference fringes oscillations with the PAOT reflectance due to interference between the reflected light from the bottom (PAOT/Al) and top (PAOT/air) interfaces. For TiO_2_/TiN-coated PAOT, the oscillation strength of the interference is stronger than PAOT. This is ascribed to the strong light modulation reflected from the top interfaces of the TiO_2_/TiN layer^36^. Therefore, the amplitudes of the reflected beams are improved.

The optical absorbance of TiO_2_ and TiO_2_/TiN is shown in Fig. 4b. From the figure, the TiO_2_ nanotube has a strong absorbance in the UV that is related to Π–Π* of the titanium ions^37,38^.

Then, the absorption decreases sharply with increase wavelengths and becomes nearly constant above 600 nm. This suggested that the TiO_2_ showed a low spectral response to the visible light. For TiO_2_/TiN film, the right absorption edge displays redshift towards a higher wavelength at the visible region compared with that of TiO_2_. Also, the absorbance is enhanced by coating these TiO_2_ nanotubes with a very thin film of TiN. Also, the absorbance is enhanced by coating these nanotubes with a very thin film of TiN.

In general, the bandgaps of TiO_2_ and TiO_2_/TiN are calculated based on the Tauc equation for direct optical band gaps of semiconductor, Eqs. 1, 2^39,40^.

1 $${(\upalpha \mathrm{h} \upnu )}^{2}=\mathrm{K }\left(\mathrm{h} \upnu -\mathrm{Eg}\right)$$

2 $${\upalpha }=\left(\frac{2.303}{\mathrm{d}}\right)\mathrm{A}$$

where Eg is the energy bandgap, h is the Planck constant, $\upnu$ is the light frequency, K is the constant, A is the optical absorbance, d is the material thickness, and ${\upalpha }$ is absorption coefficient.

From Fig. 4b, the energy gap value of TiO_2_ is decreased from 3.1 to 2.3 eV after sputtering TiN which is agreeing with the redshift of the absorption edge. This decrease is due in Eg due to increasing free carriers^20^. The TiN covalent bond enables one electron to leave the Ti atom. The barrier at the TiN/TiO_2_ interface permits free electrons to transfer from TiN to TiO2. The boundless electron needs little energy for its freedom. So, the prepared PAOT/TiO_2_/TiN materials can be applied as a photodetector, in which they have a good absorbance in the UV and initial part of Vis regions.

### Testing PAOT/TiO_2_/TiN as a photodetector

#### Effect of light power intensity

**Figure 5:**
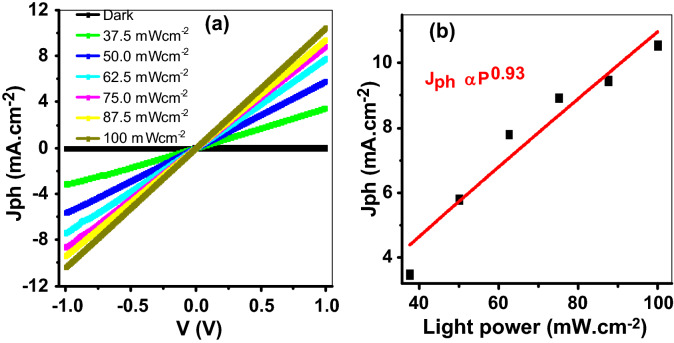
(**a**) The effect of light power density on the produced J_ph_ from − 1 to + 1 V and (**b**) the relation between the light power and the produced J_ph_ at + 1.0 V.

The dark current (J_d_) is measured and presented in Fig. 5. The value of this current is very small that changed from − 1.8 to 1.8 µA cm^−2^ under an applied potential from − 1 to + 1 V. This indicates the PAOT/TiO_2_/TiN has very small charge electrons that respond to the applied potential.

Under the dark condition, On the TiO2/TiN surface, oxygen molecules are adsorbed and free electrons from the conduction band are captured as follows, Eq. (3)^41,42^;

3 $$\frac{1}{2}{\mathrm{O}}_{2}+2{\mathrm{e}}^{2-}\to {\mathrm{O}}^{2-}$$

This results in enhanced resistivity and consequently lowers the current density. Under light illumination, electron–hole pairs are generating as a result of the electrons that will transfer from the valence band to the conduction band. The holes drift to the surface and desorb the oxygen ions according to Eqs. 4 and 5.

4 $$\mathrm{hv}\to {\mathrm{e}}^{-}+ {\mathrm{h}}^{+}$$

5 $$2{h}^{+ }+ {O}^{2-}\to O$$

The remaining electrons increase the conductivity of TiO_2_/TiN and therefore the photocurrent increases.

When the light source is switched off (dark), the adsorbed oxygen molecules on the TiO_2_/TiN surface are desorption induces the release of the electrons. Thus, the sensor reverts to its initial mode.

The behavior of oxygen atoms and molecules on the TiN surface was previously revealed in many works. Rodriguez et al. experimental studied the oxidation of TiN using synchrotron-based photoemission^43^. There was chemisorption of oxygen without significant surface oxidation at the lowest temperature. The adsorption due to van der Waals forces between the TiN adsorbing material and the adsorbed O_2_ molecules. Seifitokaldani et al. investigated the interaction between oxygen molecule and TiN surface using density functional theory (DFT)^44^. The calculated of oxygen adsorption energy proved oxygen adsorption on the TiN. Sinnott et al. examined computationally the TiN surface by using third-generation charge-optimized many-body (COMB3) potential formalism and compared with available experimental data^45^. The simulation results predict that the oxygen molecule binds initially to a Ti atom in the TiN surface. Subsequently, it moves to a bridge position over two Ti atoms and then dissociates. The dissociation of oxygen molecules is adsorbed on the TiN surface. The bridge Ti atoms is the preferred adsorption site for the oxygen molecule^46^.

The effect of light power intensity on the PAOT/TiO_2_/TiN photodetector from 37.5 to 100 mW/cm^2^ at room temperature is shown in Fig. 5. The TiN/TiO_2_ exhibit a linear I–V curve when at low voltage, which agrees with the previous work^47^.

The values of photocurrent density change greatly. Jph with the applied light power intensity. The Jph values are increased from 0.1 to 10.73 mA.cm^−2^ with increasing the light power from 37.5 to 100 mW/cm^2^. The plasmonic resonance occurring in TiN nanostructures and generating this photocurrent. The relation between the light power intensity and the produced photocurrent density at 1.0 V is mentioned in Fig. 5b. The nonlinear relation between light intensity and photocurrent density indicates the complex electron–hole transportation reaction^48^. This confirms the generation of more carriers on the material surface with increasing the light power intensity as a result of increasing excitation of electrons from VB to CB^49^.

The relation between photocurrent and light intensity can be described by a simple power-law as Eq. (6).

6 $${J}_{ph}=B{P}^{y}$$

where B is a wavelength constant, P is the incident light power. y is the exponent parameter that determines the sensitivity of photodetector (photocurrent) to the incident light intensity. This parameter refers to the complex process of electron–hole generation, recombination, and trapping of the carriers in photodetectors^50^. It can determine the response rate. By fitting the experimental results using the previous equation, red line in Fig. 5b, y is estimated to be nearly integer exponent (0.93), which suggesting highly photosensing ability^51^. These results indicate the prepared PAOT/TiO_2_/TiN can work as a good photodetector for the light power intensity.

Peng gives the linear range of dynamic (LDR, usually quoted in dB), Eq. (7)^52^:

7 $$LDR=20\mathrm{log}\frac{{J}_{ph}}{{J}_{d}}$$

At 100 mW/cm^2^, the LDR value is estimated to be 68.8 dB. The relatively large LDR indicates that the device is linearly responsive.

### Light wavelength

**Figure 6:**
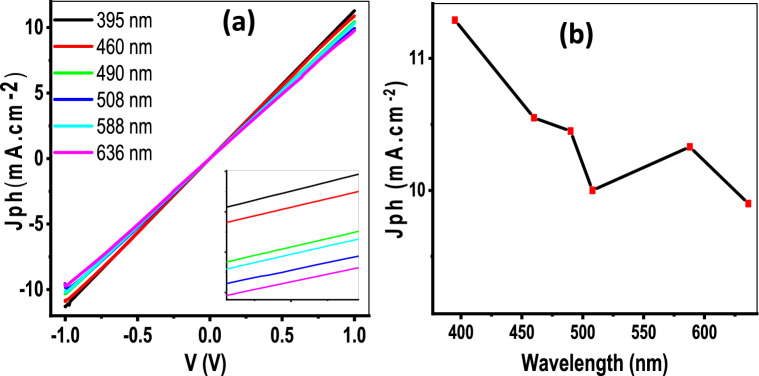
(**a**) The relation between voltage and Jph under monochromatic illuminated with different wavelengths and (**b**) V-Jph relation at 1.0 V for PAOT/TiO_2_/TiN photodetector.

Photocurrent–voltage characteristics (Jph-V) have been recorded with a series of monochromatic wavelengths to obtain the spectral response of the device PAOT/TiO_2_/TiN. Figure 6 shows J_ph_-V characteristics under monochromatic wavelength illumination ranging from UV to NIR (395–636 nm). From this figure, the Photocurrent density values decrease as the wavelengths increase from 395 to 508 nm, then increase again at 588 nm, then continue in decreasing till reach 636 nm. Figure 6b gives the Jph values at + 1 V under illumination with different monochromator light. The increase of Jph values produced by the increase in wavelengths is related to Jph unsaturated phenomena, in which increases the wavelengths causes an increase in the released photo-generated current^53,54^. This matches the optical analyses of the prepared materials, Fig. 4.

The photodetector performance is determined from the calculation of some parameters such as the photoresponsivity (R), specific detectivity (D), and external quantum efficiency (EQE)^55^. The R-value represents the relationship between the photocurrent density and the intensity of the light^56^. It can be estimated from the I–V data at + 1 V according to the following equation, Eq. (8)^1^.

8 $$\mathrm{ R}= \frac{{\mathrm{J}}_{\mathrm{ph}} - {\mathrm{J}}_{\mathrm{d}}}{\mathrm{light\, power}}$$

The D values represent the photodetector sensitivity that can be calculated depends on the R-value from Eq. (9)^24^.

9 $$\mathrm{D}=\mathrm{R }\sqrt{A /2\mathrm{ e }{\mathrm{J}}_{\mathrm{d}}}$$

where A is the effective photodetector surface area and e is the electron charge.

**Figure 7:**
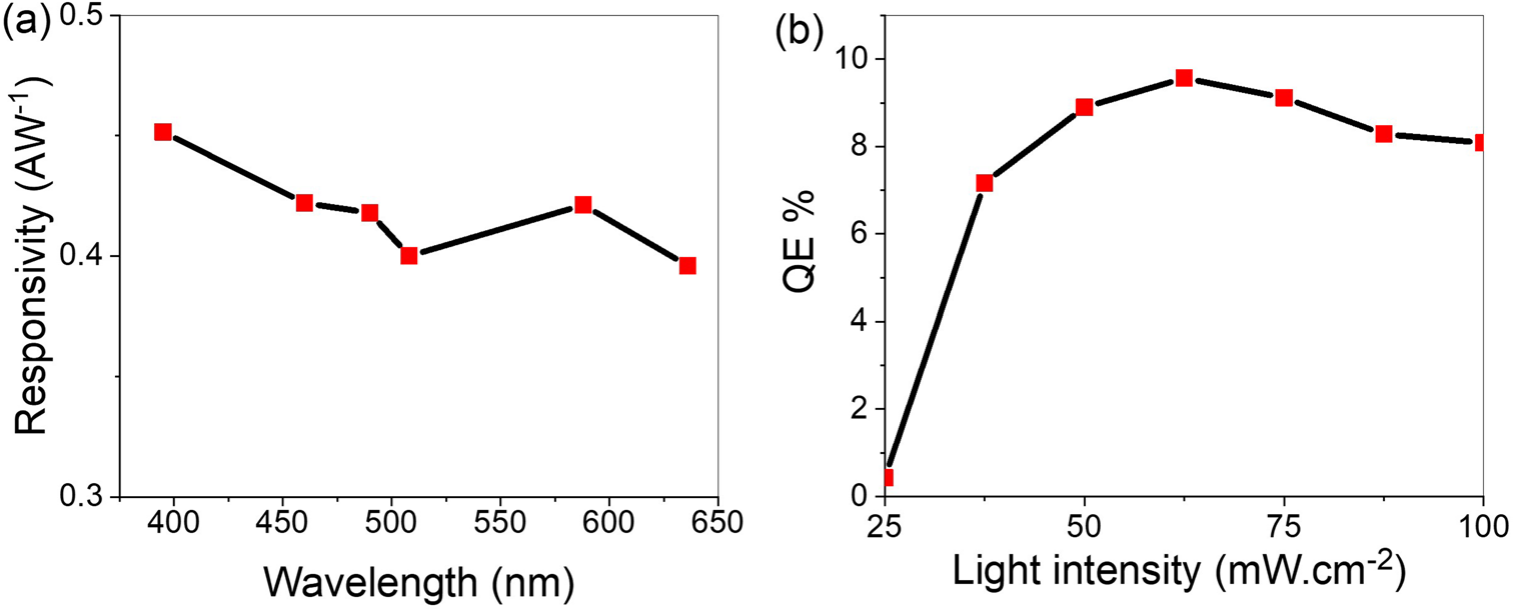
(**a**) The responsivity as a function of wavelengths and (**b**) the EQE under different intensity of light illumination.

The responsivity of the photodetector versus the applied wavelengths at 62.5 mW is shown in Fig. 7a. The maximum photoresponse is R = 450 mAW^−1^ at about 400 nm. This agrees well with the J_ph_ values as shown in Fig. 6.

The device has significant performance in the region of visible light due to the TiN plasmonic contribution in this region.

The device's specific detectivity reaches its peak value, D = 8.0 × 10^12^ Jones, at about 400 nm.

The external quantum efficiency (EQE) is the relation between the incident light photon flux and produced electrons^57^. The photon flux is directly proportional to the light intensity. The EQE value is determined from the R-value depending on the light wavelength (λ) according to Eq. (10)^58^.

10 $$\mathrm{EQE}=\mathrm{ R }\frac{1240}{\uplambda }100$$

The EQE for PAOT/TiO_2_/TiN is changed from 0.42 to 9.64% with changing the light intensity from 25 to 62.5 mW cm^-2^, respectively, and then it decreases to 8.07% as light intensity increases to 100 mW cm^-2^ as mentioned in Fig. 7b.

Based on the above results, the fabricated photodetector exhibits better performance in terms of photoresponsivity and quantum efficiency. Therefore, the prepared PAOT/TiO_2_/TiN works well as a novel photoreactor for detecting the light power intensity and wavelengths with high efficiency.

### Stability and reproducibility

**Figure 8:**
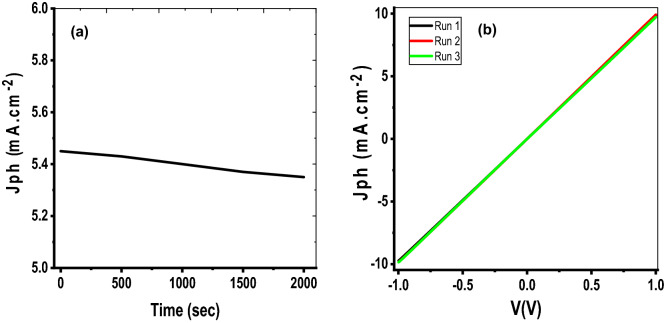
(**a**) The stability and (**b**) the reproducibility of PAOT/TiO_2_/TiN photodetector.

The stability and reproducibility of the prepared PAOT/TiO_2_/TiN photodetector are studied as shown in Fig. 8. The study of the photoelectrode stability was carried out by applying of a 1.0 V potential on the photodetector and measuring the produced J_ph_. From Fig. 8a, the electrode has high stability till 2000s. The value of Jph is nearly constant for a long period indicating that the fabricated PDs have an acceptable stability. A very small photocurrent change over time due to adsorption of O_2_ molecules on PD surface. This high stability comes from the construction of the prepared photodetector that is based on inorganic stable materials^59^.

The reproducibility measurements were carried out by testing the photoelectrode many times under light intensity 37.5 and 100 mW/cm^2^. From Fig. 8b, all the runs have almost the same values. This refers to a good reproducibility of the prepared detector and simultaneously confirms the stability of the detector^60^.

Finally, the values of R, D, and EQE of the proposed PD are higher than those previously reported as summarized in Table 1.

**Table 1:** Comparison of the performance for the prepared photodetectors with previous works.

Structure	Wavelength (nm)	Bais (V)	R (AW^−1^)	D (Jone)
TiN/TiO_2_^47^	550	5	-	6.0 × 10^10^
InTiO_2_-Ge^61^	1550	2	0.185	22 × 10^11^
Se/TiO_2_^62^	450	1	0.005	1.0 × 10^12^
TiO_2_-PANI^63^	320	0	0.003	–
TiO_2_/NiO^64^	350	0	0.0004	–
GaN ^65^	325	5	0.340	1.24 × 10^9^
Graphene/GaN^66^	365	7	0.003	1.45 × 10^10^
ZnO/GaN^67^	300	0	0.176	2.5 × 10^12^
Ag-ZnSe^68^	480	3	0.184	9.2 × 10^11^
ZnO/Ag/ZnO^69^	300	1	0.100	–
ZnO/RGO^70^	350	5	0.0013	–
MoS_2_^71^	600	5	0.120	1.0 × 10^10^
GO/Cu_2_O^72^	300	2	0.0005	1.0 × 10^6^
ZnS/Ag^73^	300	0	0.100	1.67 × 10^10^
POAT/TiO_2_/TiN (this work)	400	1	0.45	8.0 × 10^12^

## Mechanism

**Figure 9:**
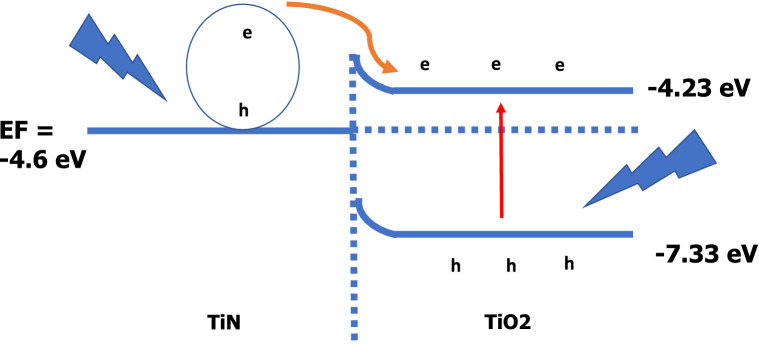
Energy level diagram showing the transportation of charge carriers for TiO_2_/TiN photodetector.

The working principle of PAOT/TiO_2_/TiN photodetector is mentioned through interaction between the photon incident, NPs and energy band theory as seen in the schematic in Fig. 9. The energy bandgap of TiO_2_ is about 3.1 eV as mentioned before in some literature with the energy diagram^50,74^. Under the light, illumination, the electron–hole pairs in TiO_2_ are generated, which contributes to the generation of photo current in the PD. Meanwhile, the electromagnetic fields in TiN can also be increased due to the electron collective oscillation as a result of surface plasmon^54^. Also, the exciting hot electrons and high light absorption of TiN enhanced the photocurrent of the PD device.

For the TiN/TiO_2_ junction, the carriers' diffusion (electrons and holes) continues until the Fermi energy (E_F_) becomes the same in both materials. This leads to band bending at the TiN/TiO_2_ interface and formed a depletion region (or built-in electric field).

The built-in electric field can efficiently separate electron–hole pairs that generated. When the TiN layer is exposed to the light, hot electrons and holes are generated at the Fermi level of TiN as a result of surface plasmon resonance. The work function of TiN (4.3 eV) is lower than that of TiO_2_ (4.9–5.5 eV). The energy difference between TiO_2_ valence band and TiN Fermi level is too high, and this prevents injection of holes from TiN to TiO_2_.

On the other hand, the barrier height between TiO_2_ conduction band and TiN Fermi level is small, so the hot electrons can be transferred from TiN to the conduction band of the TiO_2_ after passing the barrier^75^. This way prevents the carrier’s recombination which helps in the continuous electron flow into the device and leads to photocurrent generation. Also, the good compact synthesis of these two layers forms another factor for electrons flow.

## Conclusion

We have prepared a novel photodetector PAOT/TiO_2_/TiN with high stability and low cost and high efficiency for industrial application. The photodetector was tested under different light intensity (37.5 to 100 mW/cm^2^) and wavelengths (395–636 nm). The photodetector has R, D, and EQE of 450 mAW^-1^, 8.0 × 10^12^ Jones, and 9.64%, respectively. All the materials of the photodetector were prepared with highly controlled techniques. Different characterization analyses were used to confirm the morphology, chemical structure, and optical properties. We look forward to widening the application of the prepared photodetector in the space industry, light-sensing devices, and smart screens.
